# Pre-transplant Serum Procalcitonin as a Predictor of Early Post-transplant Sepsis and Mortality After Living Donor Liver Transplantation: A Prospective Observational Study

**DOI:** 10.7759/cureus.71364

**Published:** 2024-10-13

**Authors:** Sonam Satija, Lalita G Mitra, Gaurav Sindwani, Udit Dhingra, Anil Yadav, Mahesh Arora, Viniyendra Pamecha

**Affiliations:** 1 Anaesthesiology, Institute of Liver and Biliary Sciences, Delhi, IND; 2 Anaesthesiology, Critical Care and Pain, Homi Bhabha Cancer Hospital and Research Centre, New Chandigarh, New Chandigarh, IND; 3 Hepatopancretaobiliary Surgery, Institute of Liver and Biliary Sciences, Delhi, IND

**Keywords:** liver transplantation, mortality, postoperative outcomes, procalcitonin, risk factors, sepsis

## Abstract

Purpose: The early post-transplant period after liver transplantation is critical, as recipients are highly susceptible to sepsis due to their immune-compromised state. This study aimed to identify the association between preoperative procalcitonin and early post-transplant sepsis and mortality at one month after living donor liver transplantation (LDLT).

Methodology: All patients who underwent LDLT from July 2021 to December 2021 were recruited prospectively. Participants were divided into two groups based on preoperative PCT levels: elevated (>0.5 ng/ml) and low levels (<0.5 ng/ml). Serum procalcitonin (PCT) levels were measured on the day of transplant and on postoperative days 3 and 7. The relationship between preoperative PCT and post-transplant sepsis was evaluated using a Chi-square test, and receiver operating characteristic (ROC) curves were generated.

Results: Sepsis occurred in 48.3% of patients, with a significant association between elevated preoperative PCT levels and early post-transplant sepsis (p=0.023). The ROC curve for preoperative PCT showed moderate predictive ability (area under curve (AUC)=0.664), while PCT levels on postoperative day 3 demonstrated better discriminatory power (AUC=0.790). PCT levels measured on day 7 also had good diagnostic accuracy, with an AUC of 0.843 and a significant difference between the sepsis and non-sepsis groups (p=0.002). The length of ICU stay was significantly longer in the sepsis group (p=0.009).

Conclusion: Elevated preoperative PCT levels can predict early post-transplant sepsis in LDLT patients. PCT monitoring may enhance risk stratification and guide perioperative management, improving post-transplant outcomes.

## Introduction

Liver transplantation remains the definitive treatment for end-stage liver disease and acute liver failure, offering a chance for long-term survival and improved quality of life. However, the procedure presents significant perioperative challenges, including the risk of post-transplant sepsis, which are a leading cause of morbidity and mortality, especially in the first month post-transplant [1-3]. The early post-transplant period is critical, as recipients are highly susceptible to sepsis due to their immune-compromised state. This vulnerability is exacerbated by systemic inflammatory response syndrome (SIRS), triggered by non-infectious factors like surgical stress and reperfusion injury [4,5]. Also, it is very difficult to determine clinical signs of infection in these immunosuppressed patients. Microbiological culture is the gold standard test for diagnosis of sepsis but requires time, leading to substantial diagnostic and therapeutic delay. Post-transplant sepsis can progress rapidly, leading to septic shock requiring cardiopulmonary and renal replacement therapy [6,7]. So, it’s of utmost importance to timely diagnose and treat sepsis in the post-transplant period.

Procalcitonin (PCT) is one biomarker that has gained considerable attention in recognizing sepsis early. PCT levels are normally undetectable in healthy individuals but rise significantly in response to systemic infections and sepsis. PCT is more specific for bacterial infections. Various tissues, including the liver, kidney, adrenal glands, prostate glands, small intestines, and peripheral blood cells, have been shown to produce PCT. However, the liver is the most significant site for PCT production. The plasma level of PCT starts rising within six hours from an insult and reaches a plateau between eight and 24 hours, and decreases rapidly after the disappearance of an insult. This rapid kinetics makes PCT a valuable tool in the early detection of sepsis, particularly in critically ill patients [4,5].

Chronic liver disease (CLD) can affect PCT production, potentially causing lower-than-expected levels. However, clinical observations revealed that serum PCT levels may rise in the absence of bacterial infections in advanced liver failure, suggesting a more complex relationship between the liver and PCT. Despite variable thresholds, PCT remains a useful diagnostic maker for infection in CLD patients [8,9].

However, there is limited data on the role of serum PCT levels in predicting postoperative septic complications, and this is an area of interest, as it could potentially help identify patients at high risk for postoperative sepsis. Given these challenges, this study aims to identify the association between preoperative procalcitonin and early post-transplant sepsis within seven days and mortality at one month after living donor liver transplantation (LDLT).

## Materials and methods

This prospective observational study was conducted from July 2021 to December 2021 after approval from the Institutional Ethics Committee IEC/2020/73/NA05 and Clinical Trial Registry of India (CTRI/2021/03/032213), trial REF/2021/02/041256. The study enrolled all consenting patients aged 18-60 years with chronic liver disease undergoing live donor liver transplantation. Paediatric patients, pregnant patients, and those undergoing deceased donor liver transplantation (DDLT) were excluded.

Bacterial infections that occurred prior to LDLT and necessitated intravenous antimicrobial therapy during the same hospital stay were identified as pre-transplant infections. Infections were classified according to the Centers for Disease Control and Prevention (CDC) criteria and the standards applied to liver transplant recipients [10]. Post-transplant sepsis was characterized by life-threatening organ dysfunction triggered by an infection, as per the Third International Consensus Definitions for Sepsis and Septic Shock (Sepsis-3). Organ dysfunction was identified as an acute change in total Sequential Organ Failure Assessment (SOFA) score ≥2 points from baseline [11]. Early post-transplant sepsis was defined as sepsis occurring within one week after LDLT, identified by a SOFA score increase of ≥2 points.

All routine blood investigations were sent in the morning before liver transplantation to assess pre-transplant conditions. A blood sample for PCT levels under all aseptic precautions was drawn in the operating room from a peripheral cannula; 3.5 ml of blood was collected in a serum separator tube with a yellow top and sent for measurement. The PCT level is measured using the immunoassay technique, enzyme-linked immunosorbent assay (ELISA). PCT levels were measured again on days 3 and 7 post-transplant to see the trends.

All patients received standard anaesthesia care, including monitoring with ECG, non-invasive blood pressure, and pulse oximetry. Induction was performed with fentanyl, propofol, and atracurium, followed by endotracheal intubation and mechanical ventilation. Invasive vascular lines were inserted under ultrasound guidance for real-time arterial blood pressure monitoring and arterial blood gas analysis. We placed a 7 Fr sheath and a 7.5 Fr central venous catheter for fluid and drug administration. Antimicrobial prophylaxis included piperacillin-tazobactam 4.5 gm twice a day (BD), fluconazole 200 mg once a day (OD), teicoplanin 400 mg BD, and metronidazole 500 mg thrice a day (TDS), administered 45 minutes to 1 hour before surgery and continued for 72 hours post-liver transplantation (LT). We selected the right lobe graft or left lobe graft based on the results of the volumetric study. The target graft-to-recipient weight ratio (GRWR) was 0.8 or more. Fluids were administered based on goal-directed therapy depending upon the readings of advanced haemodynamic monitors such as Flotrac and transoesophageal echocardiography. Coagulation assessment was done based on point-of-care coagulation tests (TEG) at different stages of surgery and clinical haemostasis. All patients received an injection of methylprednisolone 100 mg at the time of graft implantation. Post-reperfusion syndrome was managed with fluids and vasopressors. All LT recipients were electively mechanically ventilated overnight in the transplant intensive care unit. All patients were weaned and extubated on postoperative day 1 and started with nasogastric feedings. Immunosuppression therapy in the postoperative period comprised of steroids, tacrolimus, and mycophenolate mofetil. The steroids were gradually tapered by day 3. All central venous lines and arterial catheters were removed or changed by day 6. Daily SOFA scores were calculated from day 1 to day 7, along with all routine blood investigations to look for sepsis. Microbiological cultures were sent at the suspicion of infection. All patients with post-transplant sepsis and septic shock were managed with broad-spectrum antibiotics and anti-fungal medications titrated to culture sensitivity reports. Procalcitonin levels were determined in all patients on postoperative day 3 and day 7 to see the trends. All patients were followed for 30 days.

Statistical analysis: The study enrolled 29 patients using convenience sampling based on the number of individuals presenting to our department during the study period. Data were entered into Microsoft Excel and analysed using the SPSS software, trial version 23 (IBM Corp., Armonk, NY). Continuous variables were presented as mean and standard deviation, and comparisons were made using Student's t-test or Mann-Whitney U test, as appropriate. Categorical data were compared using the Chi-square test or Fisher's exact test. Receiver Operating Characteristic (ROC) curves were generated to calculate the specificity and sensitivity of PCT levels at different time points. P-value <0.05 is considered statistically significant.

## Results

**Figure 1:**
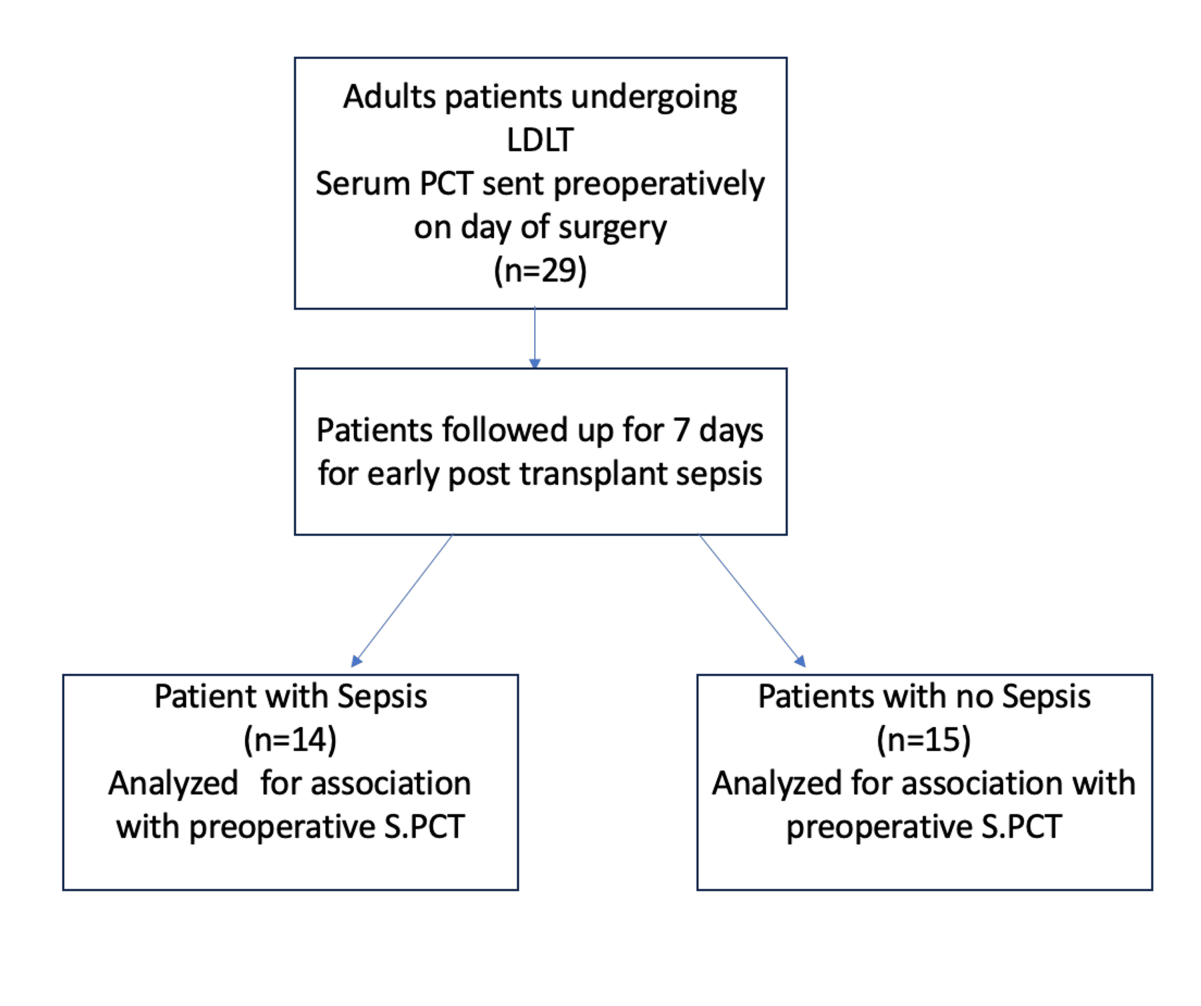
Consort flow diagram LDLT: Living donor liver transplantation; PCT: Procalcitonin; S.PCT: Serum procalcitonin.

The study enrolled 29 participants, with a gender distribution of three females (10.3%) and 26 males (89.7%) (Figure 1). The mean age of the participants was 42.52±12.28 years. The comparisons of baseline characteristics, as well as pre-operative, intra-operative, and post-operative parameters between the sepsis and non-sepsis groups, are detailed in Tables 1, 2. The mean Model for End-Stage Liver Disease (MELD) for the patients undergoing surgery was 23. Notably, 14 participants (48.3%) developed sepsis during the study period. The median SOFA score in patients at the time of sepsis was 11. Ethanol consumption was identified as the primary aetiology in both the sepsis group seven (50%) and the non-sepsis group seven (46.7%)

**Table 1:** Comparison of baseline characteristics, aetiology and risk factors in groups with and without sepsis Data represented as N (%); Chi-Square test and t-test applied, wherever applicable; * p value< 0.05 significant. ATT: Anti-tuberculosis treatment.

Parameter			Early post-transplant sepsis n=29		Total	z-value/t-value	p-value
			(+) n=14	(-) n=15			
	Baseline characteristics						
Participant distribution n (%)			14 (48.3%)	15 (51.7%)	29 (100%)		-
Age (in years) Mean±S.D.			43.64±12.77	41.47±12.17	42.52±12.28	0.47	0.642
Gender n (%)		Male	13 (92.9%)	13 (86.7%)	26 (89.7%)	0.30	1.000
		Female	1 (7.1%)	2 (13.3%)	3 (10.3%)		
	Aetiology						
Ethanol n (%)			7 (50%)	7 (46.7%)	14 (48.3%)	6.05 -	0.858
Non-Alcoholic Steatohepatitis n (%)			2 (14.3%)	1 (6.7%)	3 (10.3%)		0.501
Hepatitis B infection n (%)			1 (7.1%)	1 (6.7%)	2 (6.9%)		0.960
Hepatitis C infection n (%)			0 (0.0%)	1 (6.7%)	1 (3.4%)		0.326
ATT n (%)			0 (0.0%)	1 (6.7%)	1 (3.4%)		0.326
Citrullinemia n (%)			1 (7.1%)	0	1 (3.4%)		0.292
Cryptogenic liver cirrhosis n (%)			1 (7.1%)	2 (13.3%)	3 (10.3%)		0.584
Autoimmune Hepatitis n (%)			1 (7.1%)	2 (13.3%)	3 (10.3%)		0.584
Acute Liver Failure-Hepatitis A infection n (%)			1 (7.1%)	0 (0.0%)	1 (3.4%)		0.292
	Risk factors						
Comorbidities n (%)			5 (35.7%)	4 (26.6%)	9 (31%)	0.28	0.599
Pre-transplant ICU stay within last 6 months n (%)			2 (14.3%)	2 (13.3%)	4 (13.8%)	0.01	0.941
Pre-transplant culture positive infection within last 1 month n (%)			6 (42.8%)	6 (40%)	12 (41.3%)	0.02	0.876
Jaundice n (%)			14 (100%)	15 (100%)	29 (100%)	-	-
Pre-operative Acute Kidney Injury n (%)			5 (35.7%)	4 (26.6%)	9 (31%)	0.28	0.599
Encephalopathy n (%)			9 (64.3%)	10 (66.7%)	19 (65.5%)	0.02	0.893
Ascites n (%)			12 (85.7%)	13 (86.7%)	25 (86.2%)	0.01	0.941
Preoperative Procalcitonin (>0.5ng/ml)			6 (42.9%)	1 (6.7%)	7 (24.1%)	5.18	0.02*

**Table 2:** Comparison of pre-operative and intra-operative parameters and post-operative duration of ICU stay in groups with and without sepsis Data represented as Mean±SD; t-test applied; *p-value<0.05 significant.

Parameter		Early post-transplant sepsis		Total	t-value	p-value
		(+)	(-)			
	Pre-operative investigations					
Model for End stage Liver Disease score		22.43±8.39	23.73±4.88	23.10±6.71	0.52	0.610
Haemoglobin (in gm%)		8.86±1.44	9.54±1.40	9.21±1.43	1.30	0.206
Platelet count (per cumm)		57955.71±45095.60	61674.80±46423.75	59879.38±45003.74	0.22	0.879
Creatinine (mg/dL)		0.81±0.313	0.55±0.191	0.68±0.25	2.71	0.015*
Total Leukocyte Count (per cumm)		1161.05±2571.56	913.11±3094.40	1032.81±2806.04	0.23	0.844
Serum Bilirubin (mg/dL)		6.78±9.12	8.95±8.81	7.90±8.87	0.65	0.102
SGOT (U/L)		93.43±102.91	87.13±30.18	90.17±73.36	0.23	0.081
SGPT (U/L)		63.86±80.69	55.73±28.09	59.66±58.60	0.37	0.256
	Intra-operative findings					
Operative time (in hours)		11.86±1.41	11.27±1.98	11.55±1.72	0.92	0.366
Total fluids (in ml)		9078.57±2309.24	8113.33±2236.03	8579.31±2284.01	1.14	0.263
Number of blood products transfused		10.21±6.78	11.53±9.91	10.90±8.42	0.41	0.681
Blood loss (in m l)		3139.64±2304.42	2345.33±1582.94	2728.79±1970.16	1.08	0.591
Urine output (in ml)		988.57±474.578	1052.67±594.27	1021.72±531.24	0.32	0.752
Graft-to-recipient weight ratio (GRWR)		0.95±0.21	0.94±0.18	0.95±0.20	0.18	0.947
	Length of ICU stay					
Average length of ICU stays (in days) Mean±S.D.		16.71±6.73	10.80±4.33	13.66±6.28	3.02	0.009*

Pre-operative investigations revealed a significant difference in serum creatinine levels between the sepsis and non-sepsis groups, with higher values observed in the sepsis group (p=0.015). The length of ICU stay was also significantly longer in the sepsis group compared to the non-sepsis group (p=0.009). During the one-month follow-up period, three out of the 29 study participants (10.3%) succumbed to their conditions.

The association between preoperative procalcitonin (PCT) levels and early post-transplant sepsis is summarized in Table 3. It was observed that six (42.9%) patients who developed sepsis had preoperative PCT levels greater than 0.5 ng/ml, while only one (6.7%) patient in the non-sepsis group exhibited preoperative PCT levels above 0.5 ng/ml, a difference that was statistically significant (p=0.009).

**Table 3:** Association between preoperative procalcitonin levels and early post-transplant sepsis Data represented as N (%); Chi-square test applied; *p-value < 0.05 significant.

Pre-operative PCT level	Early post-transplant sepsis (n=29)		Total	z-value	p-value
	(+)n=14	(-)n=15			
PCT>0.5 ng/ml	6 (42.9%)	1 (6.7%)	7 (24.1%)	5.18	0.023*
PCT<0.5 ng/ml	8 (57%)	14 (93.3%)	22 (75.8%)		
Total	14 (100%)	15 (100%)	29 (100%)		

**Figure 2:**
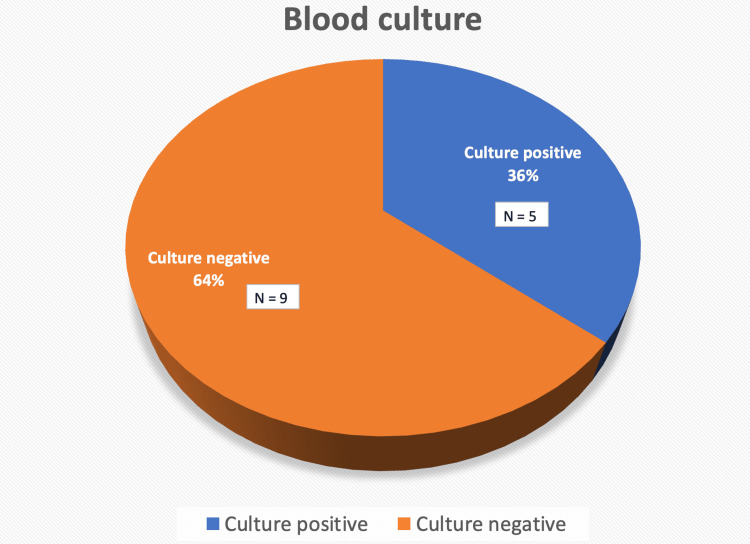
Blood culture reports of patients with sepsis Data represented as N

**Figure 3:**
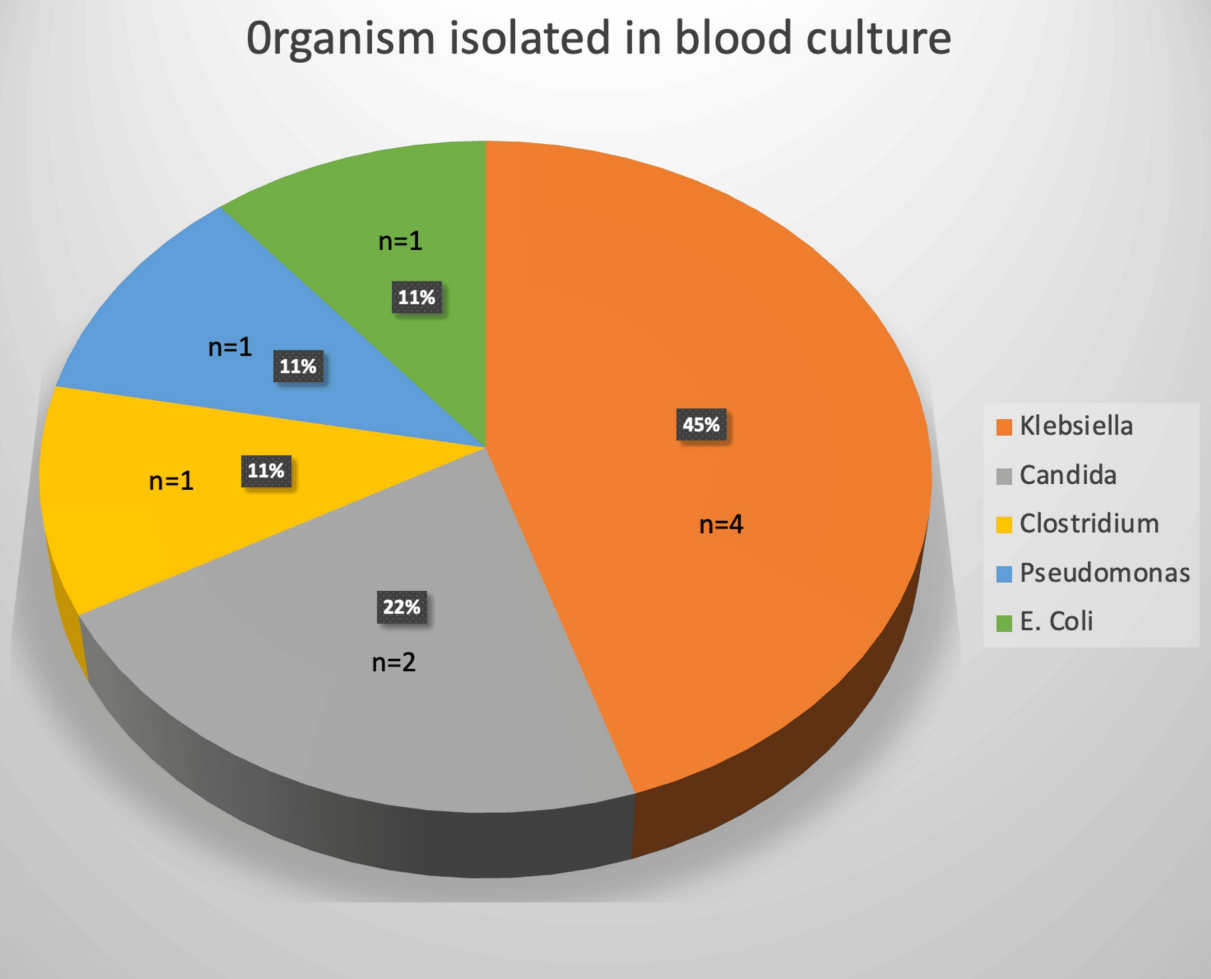
Organism isolated in blood culture Data represented as N

Figures 2, 3 illustrate the blood culture reports for patients with sepsis. Blood cultures were positive in nine (64%) out of 14 patients who developed sepsis, with *Klebsiella* being the most frequently isolated organism in four (45%), followed by *Candida* in two (22%) patients. The association between preoperative PCT levels and 30-day mortality was analysed but found to be statistically insignificant (p-value=0.473).

**Figure 4:**
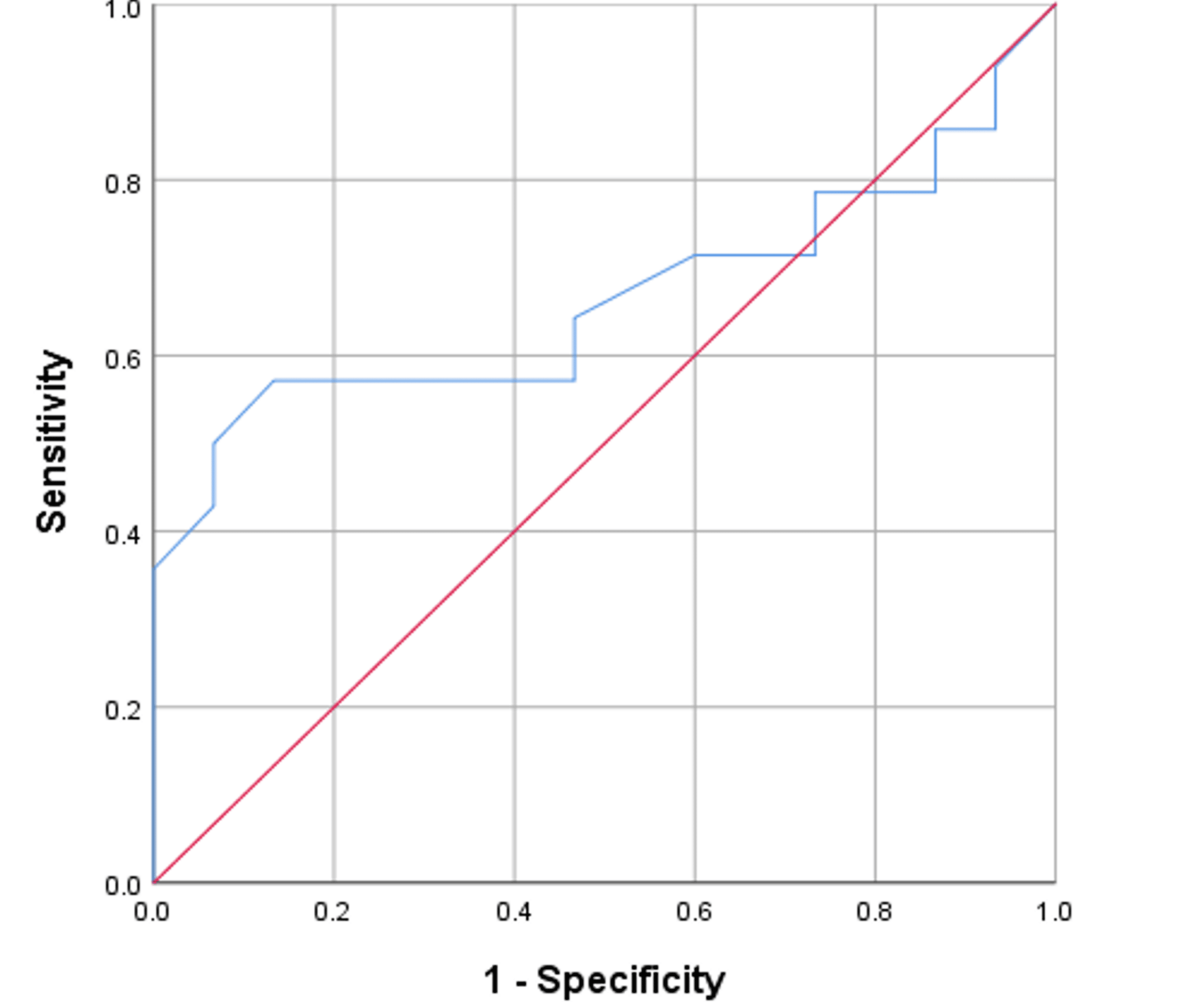
ROC curve for pre-operative PCT (PCT 0) ROC: Receiver operating characteristic; PCT: Procalcitonin.

Figure 4 shows the ROC curve for preoperative PCT levels as a predictor of early post-transplant sepsis. With an area under the curve (AUC) of 0.664, the curve indicates moderate predictive ability. The cut-off value of 0.39 ng/ml yields a sensitivity of 57.14% and a specificity of 86.67%.

**Figure 5:**
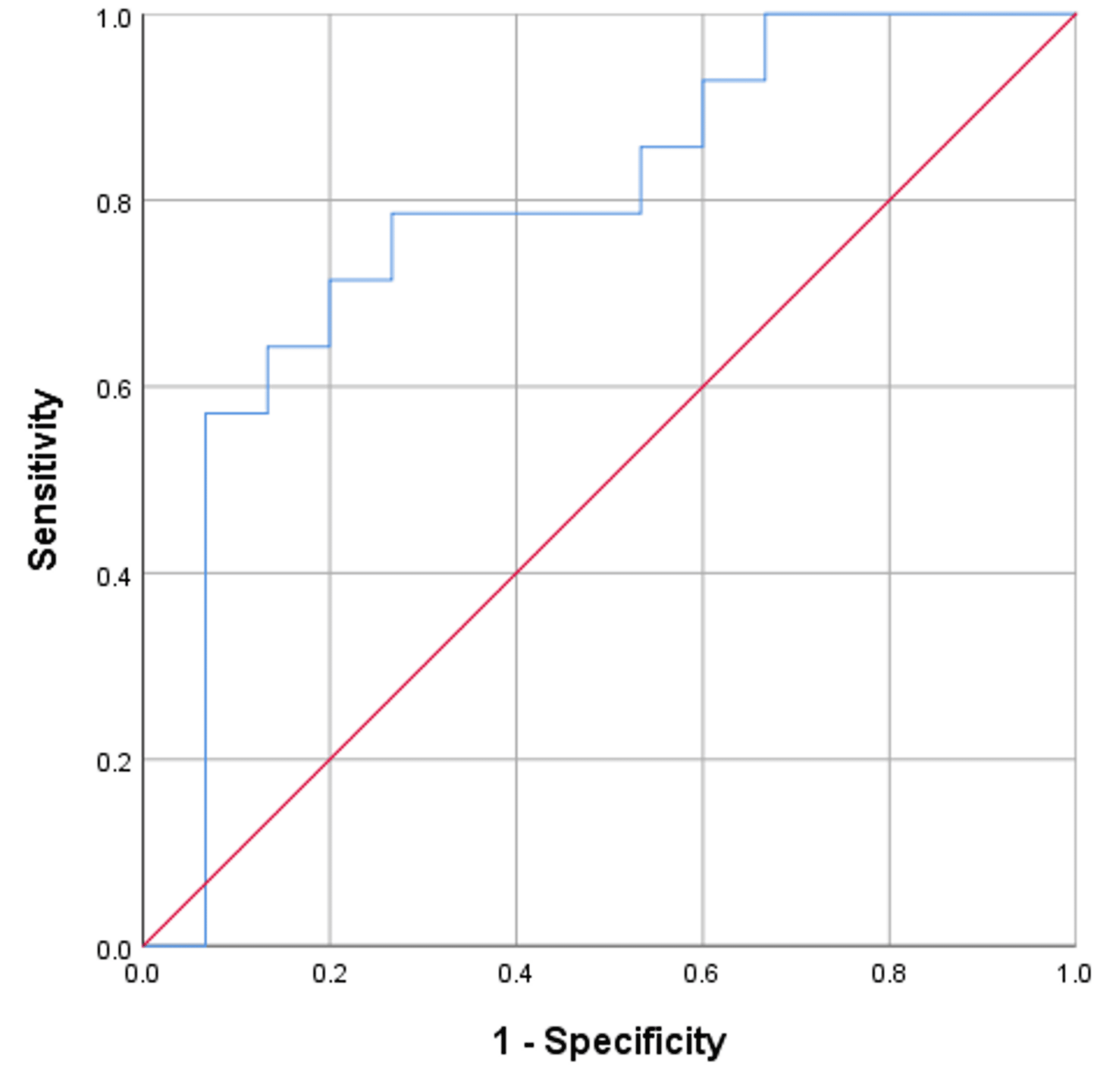
ROC curve for Post-operative day 3 PCT ROC: Receiver operating characteristic; PCT: Procalcitonin.

Figure 5 presents the ROC curve for PCT levels measured on day 3 post-transplant. The AUC is 0.790, suggesting that PCT levels on day 3 have good discriminatory power in predicting sepsis after liver transplantation. The cut-off value for PCT on day 3 is identified as 8.01 ng/ml, with a sensitivity of 78.57% and a specificity of 73.33%. These values indicate that patients with PCT levels above this threshold on day 3 are more likely to develop sepsis, while those with lower levels are less likely to do so.

**Figure 6:**
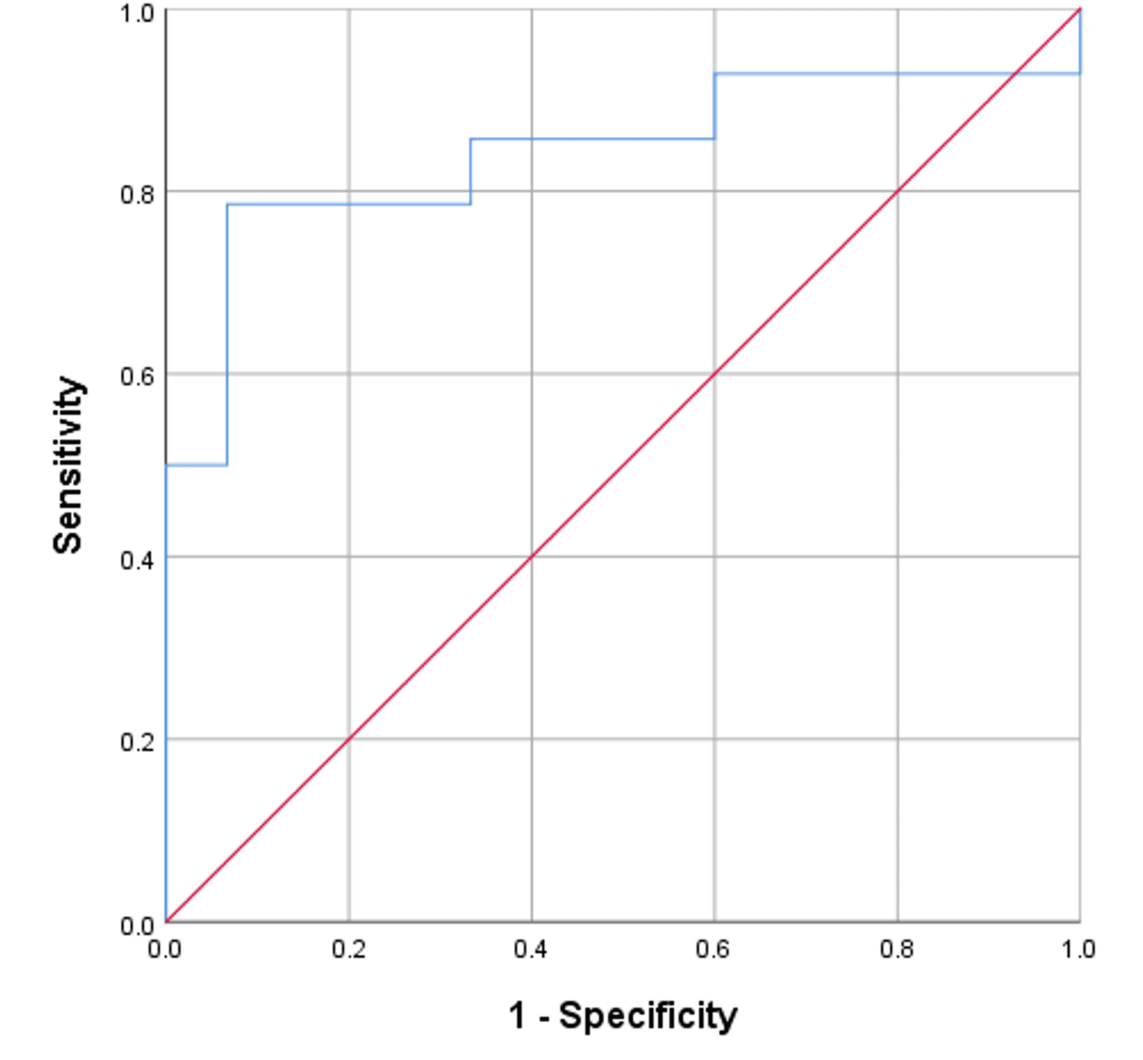
ROC curve for Post-operative day 7 PCT ROC: Receiver operating characteristic; PCT: Procalcitonin.

The ROC curve in Figure 6 yielded an AUC of 0.843, indicating good diagnostic accuracy. At a cut-off value of 1.79, the sensitivity was 78.57% and the specificity was 93.33%, with a statistically significant p-value=0.002, supporting the reliability of this cut-off point in the clinical context.

**Figure 7:**
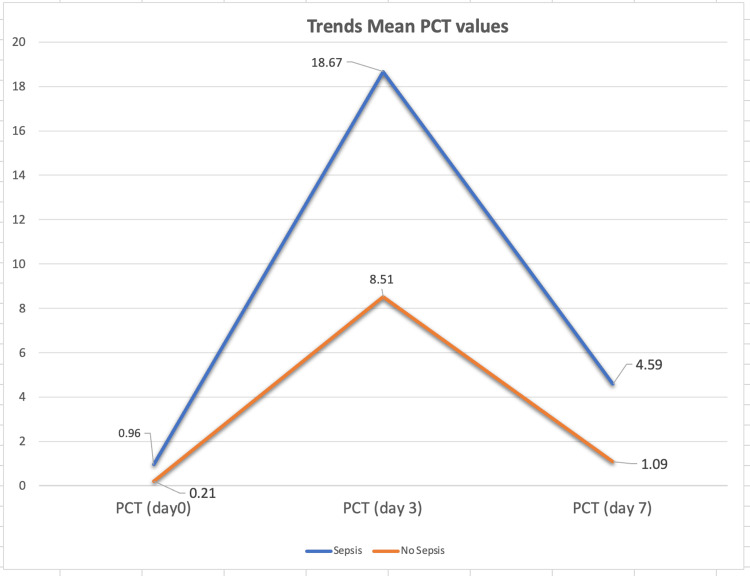
Graph showing trends of PCT in sepsis and non sepsis group Data presented as Mean ± SD. PCT: Procalcitonin.

Figure 7 demonstrates the trend of post-operative procalcitonin levels in both the sepsis and non-sepsis groups. Procalcitonin levels peaked on day 3 in all patients, with a significant difference between the sepsis and non-sepsis groups (p-value=0.024). By day 7, PCT levels had decreased from the day 3 peak, though the mean levels remained higher in the sepsis group compared to the non-sepsis group (p-value=0.020).

## Discussion

Patients with end-stage liver disease (ESLD) are predisposed to septic complications due to factors such as immunosuppression, sarcopenia, and malnutrition, as well as the invasiveness of liver transplantation. Post-transplant sepsis, defined based on the SIRS criteria, has been reported with an incidence ranging from 13% to 46% [2,5-7]. In our study, we observed an incidence of early post-transplant sepsis of 48.3%, with a corresponding mortality rate of 10.3%, which aligns with previously reported data. Despite the high risk of sepsis in this population, preventing post-transplant infections remains challenging. Biomarkers such as procalcitonin (PCT), total leukocyte count, and C-reactive protein (CRP) have been studied for their role in diagnosing and monitoring sepsis, but the association between preoperative serum PCT and post-transplant sepsis has not been well explored. We aimed to investigate whether elevated pre-transplant PCT levels (>0.5 ng/ml) could predict the development of post-transplant infections.

PCT, a 116-amino acid pro-peptide precursor of calcitonin, is predominantly produced by the thyroid gland. During systemic inflammation, it is also released from peripheral blood monocytes and macrophages [12,13]. PCT is considered a reliable marker for diagnosing infections, assessing their severity, and monitoring the progression of sepsis in various patient populations [14,15]. Chronic liver disease (CLD) can affect PCT production, potentially leading to lower-than-expected levels. However, clinical observations have shown that serum PCT levels may rise in the absence of bacterial infections in advanced liver failure, suggesting a more complex relationship between the liver and PCT. Despite this, PCT remains a useful diagnostic marker for infection in CLD patients. Our study found a significant association between preoperative PCT levels greater than 0.5 ng/ml and post-transplant sepsis (p=0.009). Patients with elevated preoperative PCT levels had a 10.5 times higher risk of developing sepsis (95% CI) compared to those with levels below 0.5 ng/ml. Elevated preoperative PCT may indicate incomplete recovery from pre-transplant infections or the presence of a latent systemic infection without overt symptoms, serving as a potential red flag. Sun et al. reported that 32% of deceased donor liver transplant (DDLT) recipients had pre-transplant infections, but adequately treated infections did not adversely impact outcomes [16]. At our institution, the protocol is to administer culture-based antibiotics and delay surgery until cultures are negative. Our findings suggest the need to reassess the criteria for determining full recovery and the timing of liver transplantation. We propose that PCT levels in the pre-transplant period could serve as a valuable marker for evaluating recovery from infection. Monitoring PCT trends could provide further insight into the patient’s condition, and a cut-off level of 0.5 ng/ml could be used to guide the timing of surgery.

Post-transplant, we observed that PCT levels peaked on postoperative day (POD) 3 and gradually declined, consistent with previous studies [5,17-19]. The exact mechanism behind this transient elevation of PCT in the immediate postoperative period remains unclear. Some researchers suggest that PCT production in the newly transplanted liver may briefly increase due to direct endotoxin entry into systemic circulation from the intestines during the anhepatic phase or a temporary reduction in the graft’s ability to clear endotoxins [20-22]. In our study, mean PCT levels were significantly higher in patients who developed sepsis compared to those who did not. Additionally, the length of hospital stay was significantly longer in the sepsis group. We did not find an association between elevated preoperative PCT and 30-day mortality post-LT, likely due to the small sample size of our study.

The major causes of post-transplant sepsis in our cohort were bloodstream infections and respiratory infections. Bloodstream infections were primarily due to prolonged venous catheterization, while respiratory infections were likely due to sarcopenia, which impairs muscle strength and vital capacity, making it difficult for patients to clear secretions and predisposing them to infections. Analysis of patients with pre-transplant infections within one month revealed that spontaneous bacterial peritonitis (SBP) was the most common pre-transplant infection, followed by pneumonia and urinary tract infections. Strategies to reduce morbidity and mortality associated with post-transplant sepsis include identifying and optimizing all risk factors before and during transplant surgery. Factors such as ABO incompatibility, impaired kidney function, Child-Pugh class C, older donor age, and massive bleeding have been previously highlighted as risk factors [4,23,24]. Our study also identified pre-transplant creatinine as an independent risk factor, underscoring that even seemingly normal creatinine levels (i.e., <1 mg/dL) can be concerning in the context of sarcopenia. It is well-documented that the estimated glomerular filtration rate (eGFR) is a more reliable measure of renal function than serum creatinine in these malnourished patients.

Limitations

The major limitations of our study include the small sample size, which may have limited the statistical power and generalizability of the results. Additionally, the diagnosis of sepsis was based exclusively on the SOFA score, which, while useful, may not fully capture the complexity of sepsis in this patient population. Furthermore, our study focused solely on early post-transplant sepsis within the first seven days, potentially overlooking cases that develop later.

## Conclusions

This study highlights the importance of pre-transplant serum procalcitonin (PCT) as a predictive biomarker for early post-transplant sepsis in living donor liver transplantation (LDLT). While pre-operative PCT predicted it, post-operative PCT on day 3 and day 7 also correlated very well with the sepsis, making this an important investigation in the early post-transplant period also. By focusing on the critical pre-operative period, the research underscores the potential of PCT to enhance clinical decision-making and improve patient outcomes. As the demand for liver transplants grows and patient care becomes increasingly complex, identifying reliable predictors of post-operative complications is crucial. This study takes a significant step towards a more personalized approach to liver transplantation, where pre-transplant biomarker assessment could inform tailored interventions, ultimately reducing the risk of post-operative sepsis and improving survival rates.

## References

[1] Kaido T, Egawa H, Tsuji H, Ashihara E, Maekawa T, Uemoto S (2009). In-hospital mortality in adult recipients of living donor liver transplantation: experience of 576 consecutive cases at a single center. Liver Transpl.

[2] Saner FH, Olde Damink SW, Pavlakovic G (2008). Pulmonary and blood stream infections in adult living donor and cadaveric liver transplant patients. Transplantation.

[3] Garbino J, Romand JA, Pittet D, Giostra E, Mentha G, Suter P (2005). Infection and rejection in liver transplant patients: a 10-year Swiss single-centre experience. Swiss Med Wkly.

[4] Kaido T, Ogawa K, Fujimoto Y, Mori A, Hatano E, Okajima H, Uemoto S (2014). Perioperative changes of procalcitonin levels in patients undergoing liver transplantation. Transpl Infect Dis.

[5] van den Broek MA, Olde Damink SW, Winkens B, Broelsch CE, Malagó M, Paul A, Saner FH (2010). Procalcitonin as a prognostic marker for infectious complications in liver transplant recipients in an intensive care unit. Liver Transpl.

[6] Ikegami T, Shirabe K, Yoshiya S (2012). Bacterial sepsis after living donor liver transplantation: the impact of early enteral nutrition. J Am Coll Surg.

[7] Takeda K, Sawada Y, Kumamoto T, Tanaka K, Endo I (2016). Severe sepsis after living donor liver transplantation: risk factors and outcomes. Transplant Proc.

[8] Lee SO, Kang SH, Abdel-Massih RC, Brown RA, Razonable RR (2011). Spectrum of early-onset and late-onset bacteremias after liver transplantation: implications for management. Liver Transpl.

[9] Dong R, Wan B, Lin S (2019). Procalcitonin and liver disease: a literature review. J Clin Transl Hepatol.

[10] Garner JS, Jarvis WR, Emori TG, Horan TC, Hughes JM (1988). CDC definitions for nosocomial infections, 1988. Am J Infect Control.

[11] Singer M, Deutschman CS, Seymour CW (2016). The third international consensus definitions for sepsis and septic shock (Sepsis-3). JAMA.

[12] Assicot M, Gendrel D, Carsin H, Raymond J, Guilbaud J, Bohuon C (1993). High serum procalcitonin concentrations in patients with sepsis and infection. Lancet.

[13] Brunkhorst FM, Heinz U, Forycki ZF (1998). Kinetics of procalcitonin in iatrogenic sepsis. Intensive Care Med.

[14] Tang BM, Eslick GD, Craig JC, McLean AS (2007). Accuracy of procalcitonin for sepsis diagnosis in critically ill patients: systematic review and meta-analysis. Lancet Infect Dis.

[15] Uzzan B, Cohen R, Nicolas P, Cucherat M, Perret GY (2006). Procalcitonin as a diagnostic test for sepsis in critically ill adults and after surgery or trauma: a systematic review and meta-analysis. Crit Care Med.

[16] Sun HY, Cacciarelli TV, Singh N (2010). Impact of pretransplant infections on clinical outcomes of liver transplant recipients. Liver Transpl.

[17] Eyraud D, Ben Ayed S, Tanguy ML (2008). Procalcitonin in liver transplantation: are high levels due to donors or recipients?. Crit Care.

[18] Zazula R, Prucha M, Tyll T, Kieslichova E (2007). Induction of procalcitonin in liver transplant patients treated with anti-thymocyte globulin. Crit Care.

[19] Perrakis A, Stirkat F, Croner RS (2016). Prognostic and diagnostic value of procalcitonin in the post-transplant setting after liver transplantation. Arch Med Sci.

[20] Fazakas J, Gondos T, Varga M, Sarvary E, Horovitz P, Perner F (2003). Analysis of systemic and regional procalcitonin serum levels during liver transplantation. Transpl Int.

[21] Kretzschmar M, Krüger A, Schirrmeister W (2003). Hepatic ischemia-reperfusion syndrome after partial liver resection (LR): hepatic venous oxygen saturation, enzyme pattern, reduced and oxidized glutathione, procalcitonin and interleukin-6. Exp Toxicol Pathol.

[22] Kornberg A, Grube T, Wagner T (2000). Differentiated therapy with prostaglandin E1 (alprostadil) after orthotopic liver transplantation: the usefulness of procalcitonin (PCT) and hepatic artery resistive index (RI) for the evaluation of early graft function and clinical course. Clin Chem Lab Med.

[23] Kim SI, Kim YJ, Jun YH (2009). Epidemiology and risk factors for bacteremia in 144 consecutive living-donor liver transplant recipients. Yonsei Med J.

[24] Iida T, Kaido T, Yagi S (2010). Posttransplant bacteremia in adult living donor liver transplant recipients. Liver Transpl.

